# Multiscale homogenization of aluminum honeycomb structures: Thermal analysis with orthotropic representative volume element and finite element method

**DOI:** 10.1016/j.heliyon.2024.e24166

**Published:** 2024-01-08

**Authors:** Ali Al-Masri, Khalil Khanafer, Kambiz Vafai

**Affiliations:** a Scientific Research Center, Australian University, Kuwait; b College of Innovation and Technology, Mechanical Engineering, University of Michigan, Flint, MI 48502, USA; c Mechanical Engineering Department, University of California, Riverside, CA 92521, USA

**Keywords:** Homogenization, Honeycomb, Thermal, Finite element, And RVE

## Abstract

This study develops a thermal homogenization model for an aluminum honeycomb panel using the representative volume element (RVE) concept, considering the orthotropic nature of the structure. The RVE thermal homogenization method is a numerical approach for analyzing heterogeneous materials. It employs a constitutive model based on RVE performance to represent thermal behavior. Effective parameters are determined through averaging techniques, and the finite element method solves the thermal problem, accounting for structure topology and material behavior. The resulting heat conduction problem is solved using the finite element method (FEM) to evaluate the effective thermal characteristics. A 3D RVE is generated based on the honeycomb panel's geometry, evaluating thermal conductivity tensor and describing the medium's thermal performance. Numerical tests validate the model by comparing it with the real honeycomb structure under sinusoidal heat flux. Results show good correlation, with maximum temperatures of 1101.9 °C in the real structure and 1096.4 °C in the medium. The homogeneous medium is further used to investigate thermal performance under convective conditions with varying panel thicknesses, achieving over 77 °C temperature reduction with the thickest panel. Natural vibration behavior is considered, demonstrating strong correlation between modal responses and natural frequencies. This modeling approach efficiently analyzes thermal behavior in large honeycomb structures, reducing computational time significantly.

## Nomenclature

c_p_: specific heat capacity
g_i_: component of the temperature gradient in i-direction, i = x, y, z
[k]: thermal conductivity tensor
[k^eff^]: effective thermal conductivity tensor
{q}: heat flux vector
{$\bar{q}$}: average heat flux vector
$\bar{\nabla T}$: average temperature gradient
T: temperature
V: volume

**Greek symbols**
[α]: thermal diffusivity tensor
ρ: density

**Abbreviations**
FE: Finite Element
FEM: Finite Element Method
RVE: Representative Volume Element

## 1 Introduction

Structured periodic materials consist of repeating unit cells, interconnected to form a strong and lightweight lattice-like framework. Honeycomb structure with its unique geometry provides exceptional strength-to-weight ratios, making them ideal for various applications [1,2]. Whether in aerospace engineering, automotive, rail vehicle or construction, honeycomb structures offer remarkable strength, durability, and efficiency. The empty spaces within the honeycomb cells minimize material usage while maintaining structural integrity, resulting in reduced weight and increased load-bearing capacity. This design not only maximizes strength but also enhances energy absorption, thermal insulation, and acoustic properties [[3], [4], [5], [6], [7], [8]]. Honeycomb structures with aluminum core material have various thermal applications due to their unique properties. They are used in heat exchangers and in cooling of electronic devices, solar panels. Moreover, honeycomb aluminum structures can be utilized for thermal energy storage [[9], [10], [11], [12]]. By incorporating phase-change materials (PCM) within the honeycomb cells, heat can be stored and released as the PCM changes phase. This application is beneficial for applications requiring thermal energy storage, such as solar thermal systems and waste heat recovery [[10], [11], [12], [13], [14], [15], [16]]. Moreover, honeycomb aluminum structures can be employed as a core material in thermal insulation systems. The air-filled cells of the honeycomb structure provide excellent insulation properties, reducing heat transfer by conduction. These structures are used in applications like building insulation panels, as well as in aerospace, automotive and rail vehicles [[17], [18], [19], [20], [21], [22], [23], [24], [25]].

Honeycomb aluminum structures are widely used in the aerospace industry for thermal management. They are employed in aircraft structures to provide lightweight heat shields, thermal barriers, and insulation in areas prone to high temperatures, such as engines and exhaust systems. Aluminum honeycomb structures are also known for their excellent fire-retardant properties and improved thermal performance [[26], [27], [28], [29], [30], [31], [32]]. Modeling the thermal behavior of honeycomb panels is a complex and critical process in various engineering applications. Honeycomb structures, composed of a series of interconnected cells, exhibit unique thermal characteristics due to their geometric arrangement. To accurately capture their thermal behavior, sophisticated numerical methods such as FEM are employed [[33], [34], [35], [36], [37], [38]]. Due to hardware limitations, the analysis of structured materials with intricate geometries can be challenging, demanding the simplification of cellular material structures. To address this challenge, researchers and engineers have explored the concept of thermal homogenization, which allows for the estimation of macroscopic thermal properties by modeling the structure as an equivalent homogeneous material. This approach enables efficient computational analysis while capturing the essential thermal characteristics of the complex honeycomb structure [[39], [40], [41], [42], [43], [44]].

Homogenization is a mathematical and computational technique used to analyze structured materials because it allows for the effective analysis of their macroscopic behavior. The structured material can be quite complex, consisting of various shapes, sizes, and orientations. Analyzing the behavior of such materials at a repeating unit cell level can be extremely challenging and computationally expensive. Homogenization provides a way to simplify this analysis by considering the material as an equivalent homogeneous material with effective properties [[45], [46], [47], [48]]. The basic idea behind homogenization is to average the properties of the different components or phases of the structured material over the RVE. This RVE is chosen to be large enough to contain a statistically representative sample of the microstructure but small enough to be considered homogeneous. By averaging the properties, such as elastic modulus, thermal conductivity, or electrical conductivity, over the RVE, the structured material can be replaced with an equivalent homogeneous material that exhibits similar macroscopic behavior [49,50] Homogenization techniques can be applied at different scales, such as the microscopic (e.g., unit cell methods), mesoscopic (e.g., Mori-Tanaka method), or macroscopic (e.g., finite element method) scales, depending on the level of detail required in the analysis. These techniques allow for the estimation of effective material properties and the prediction of the overall behavior of the structured material under different loading or environmental conditions [51,52]. By using homogenization, engineers and researchers can gain valuable insights into the mechanical, thermal, or electrical behavior of structured materials without the need for extensive and computationally expensive microstructural simulations. This simplification enables the design and optimization of materials with desired properties, leading to the development of improved and tailored materials for specific applications [53,54].

Different modeling techniques can be found in the literature with simplification approaches of the honeycomb unit cell to describe the effective material characteristics. The used models can be 0D, 1D and 2D [55]. The application of RVE for thermal homogenization of honeycomb panels has been a relatively limited area of research, with only a few researchers exploring its potential. Besides, less effort is given to the investigation of the 3D anisotropic behavior of these structures. Further research in this area has the potential to unlock new insights and enhance the design and optimization of honeycomb panels for enhanced thermal efficiency. This study focuses on the thermal homogenization of a honeycomb structure using the 3D RVE concept. The resulting thermal problem is to be solved using the FEM. The aim is to develop a constitutive model that describes the 3D thermal behavior of a homogeneous medium which is representative for the actual honeycomb structure. The obtained effective thermal characteristics, including the thermal conductivity tensor, heat capacity, and density are assigned to the homogeneous medium to accurately predict and understand the macroscopic transient thermal behavior of the honeycomb structure under different boundary condition. In addition, the material non-linearity is considered in the analysis by implementing the temperature dependent material characteristics in the model. By integrating the RVE model within the FEM software (ANSYS), we can accurately capture the intricate thermal behavior of the aluminum honeycomb structure at the macroscopic level.

### Thermal behavior of honeycomb structure

The heat transfer problem in a honeycomb structure is governed by the heat conduction equation, which takes the general form of Eq. (1)

(1) $$\nabla \cdot \left\{q\right\}=\rho c_{p}\frac{\partial T}{\partial t}$$

The left-hand side represents the divergence of the heat flux vector, where *∇* is the gradient operator. The right-hand side defines the change of internal energy density with time. *ρ* the mass density, *c*_*p*_ the specific heat capacity and the last term represents the time rate of change of the temperature (*T*).

The solution of the problem requires the additional constitutive model describing the relationship between the heat flux vector and the temperature gradient, which is given by Fourier's law, Eq. (2), as

(2) {q}=−[k]∇T

The thermal conductivity matrix *[k]* contains the thermal conductivities of the material and their number reduces to one value in the isotropic case.

Substituting equation (2), (1) leads to Eq. (3), which is the heat conduction equation in the general form.

(3) $$\nabla \cdot \left\{\left[k\right]\nabla T\right\}+\rho c_{p}\frac{\partial T}{\partial t}=0$$

The material properties in equation (3) can be combined in the form given by Eq. (4):

(4) $$\frac{1}{\rho c_{p}}\left[k\right]=\left[\alpha \right]$$

which, defines the thermal diffusivity tensor *[α]* of the medium. The thermal diffusivity tensor captures the anisotropic nature of the material, with different diffusivity values along each principal axis. The components of this tensor characterize the time-scale for penetration of a thermal front through a material layer and direction of given thickness. Furthermore, for most heat management issues in sandwich applications involving metal cores, the thermal model neglects the internal convection and radiation contribution while considering the core material's thermal conductivity to be significantly greater than that of the air or fluid within the pores. These assumptions are generally applicable in the studied case, where aluminum is the used core material [18,24,47,56]. The solution of the thermal problem can be obtained numerically using FEM, which requires the discretization of the geometry of the structure into finite elements. The FE simulation involves the solution of a nonlinear problem with Newton-Raphson solvers, which is accomplished using an arc-length method.

**Fig. 1:**
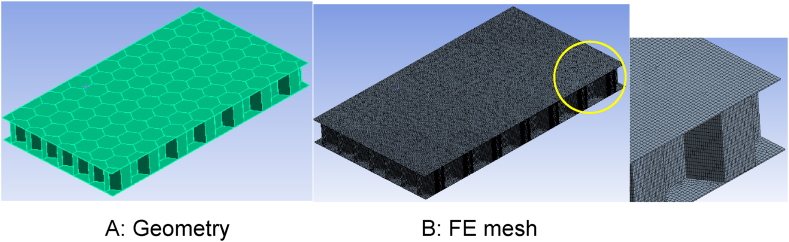
(A) Honeycomb geometry and (B) computational FE mesh.

Depending on the geometrical dimensions of the repeating unit cell in the structured medium and the extent of the considered body, a large number of elements may be needed to describe the physical behavior of the medium. An example is presented Fig. 1 (A, B), showing the model geometry and FE mesh of a honeycomb structure.

The size of the displayed body is *105 × 60.6 × 10 mm³*, and as can be seen in the right side of the figure, the FE mesh has *494263* elements and *464965* nodes. Due to the enormous computing cost, this modeling approach is not feasible in practice. However, accurately predicting the overall thermal behavior of such structures can be challenging due to their complex geometry and internal structure. Due to this fact, homogenization methods are utilized to model the thermal performance of these structures. This topic will be discussed in the next section.

## 2 RVE based homogenization

**Fig. 2:**
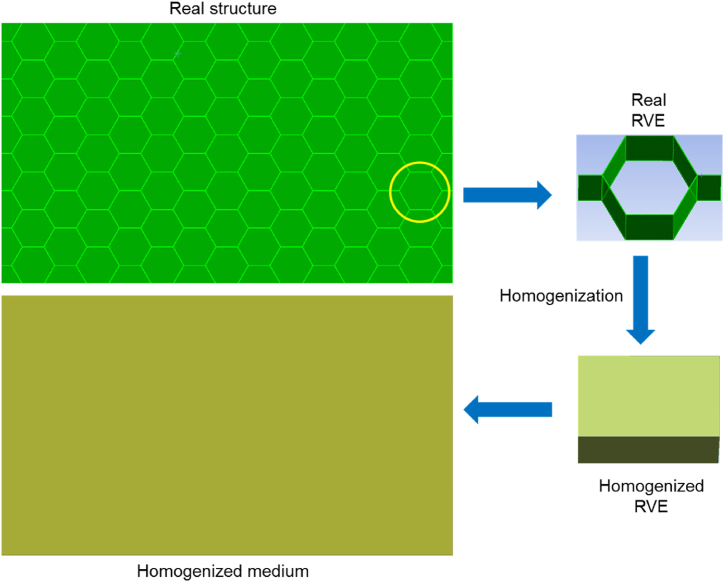
RVE based homogenization.

Thermal homogenization is a concept used to simplify the analysis of heat transfer in complex structures by replacing them with an equivalent homogeneous material. The thermal homogenization based on the RVE method is a mathematical technique used to determine the effective thermal properties of a periodic structure such as honeycomb or a composite material with a periodic array of fibers or textile fabric in a matrix. RVE homogenization methods are used in computational analysis to investigate the behavior of heterogeneous materials at the macroscopic scale by considering their structure at a lower level like meso or micro scale. These methods aim to approximate the macroscopic behavior of the material by simulating the response of an RVE, which is a small but statistically representative portion of the material. The principle is displayed in Fig. 2.

There are several multiscale RVE homogenization methods commonly used in the fields of computational physics. A few examples are: Eshelby's method, Mori-Tanaka method, Voigt-Reuss-Hill averaging method and finite element method [57]. The choice of method depends on the nature of the problem, the complexity of the microstructure, and the available computational resources. In this study the FEM based RVE homogenization method is utilized taking into account the symmetry of the honeycomb structure. In order to homogenize the 3D thermal conductivity of a honeycomb structure using the FEM-based approach, the subsequent steps are followed in the analysis.

• Definition of the RVE, including geometry, material and FE mesh generation.
• Boundary conditions.
• Solution of the FEM model.
• Post-processing.
• Calculation of the effective thermal characteristics.

## 3 Definition of the RVE: geometry and constitutive model

**Fig. 3:**
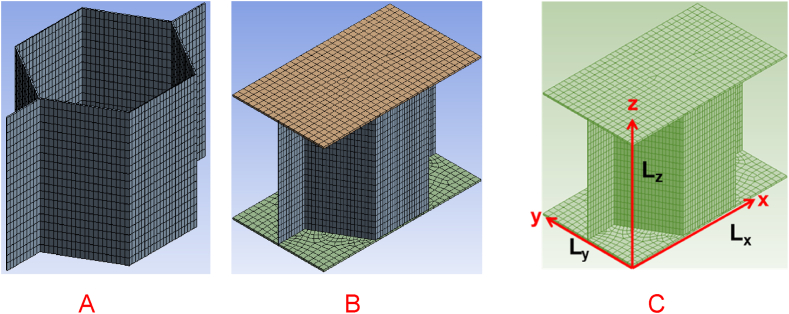
FEM of RVE, A: without skin, B: with skin, C: geometry.

The FEM of the honeycomb RVE is defined as displayed in Fig. 3 (A-C). The computational domain contains a high conductive metal, and a very low conductive air. In this case only the contribution of the solid part is considered to characterize the thermal behavior of the RVE [58]. In this study, thermal performance of a sandwich honeycomb panel is considered. For this purpose, two additional skin layers are included in the RVE model, as presented in the figure.

The analysis aims to define the properties of a homogenized medium that accurately represents the thermal response of an actual honeycomb structure. The thermal behavior of the uniform medium is determined by a constitutive model, which establishes the connection between heat flow and temperature at each material point on this scale. The physical properties within the material model are the averaged values of the respective properties in the RVE region. Typically, the constitutive model can be represented by Fourier's law in the general form of Eq. (5) as [[59], [60]].

(5) $$\left\{\bar{q}\right\}=-\left[k^{eff}\right]\bar{\nabla T}$$

where the fields of average heat flux ($\left\{\bar{q}\right\}$) and average temperature gradient ($\bar{\nabla T}$) are related by introducing the tensor of effective thermal conductivity ($\left[k^{eff}\right]$), which accounts for the anisotropy of the homogenized material. The quantities introduced in the constitutive model are defined by Eqs. (6), (7), (8):

(6) $$\left\{\bar{q}\right\}=\left\{\begin{array}{c} {\bar{q}}_{x}\\ {\bar{q}}_{y}\\ {\bar{q}}_{z} \end{array}\right\}$$

(7) $$\bar{\nabla T}=\left\{\begin{array}{c} \bar{\partial T/\partial x}\\ \bar{\partial T/\partial y}\\ \bar{\partial T/\partial z} \end{array}\right\}$$

Furthermore, due to the symmetry of the honeycomb RVE, the thermal conductivity tensor of the homogeneous medium is orthotropic and has only diagonal elements according to the equation:

(8) $$\left[k^{eff}\right]=\left[\begin{array}{ccc} k_{xx}^{eff} & 0 & 0\\ 0 & k_{yy}^{eff} & 0\\ 0 & 0 & k_{zz}^{eff} \end{array}\right]$$

The subscripts *x*, *y*, and *z* indicate the symmetrical planes of the RVE and at the same time the directions of the principal axes of the material in the homogeneous body. The averaged quantities of heat flux and temperature in Eqs. (9), (10), (11), (12) describe the thermal behavior of the homogeneous medium and they are defined by the following relationships.

(9) $${\bar{q}}_{i}=\frac{\int_{V}q_{i}dV}{\int_{V}dV},i=x,y,z$$

(10) $$\bar{T}=\frac{\int_{V}TdV}{\int_{V}dV}$$

(11) $$\bar{\nabla T}=\frac{\int_{V}\nabla TdV}{\int_{V}dV}=\frac{1}{\int_{V}dV}\left\{\begin{array}{ccc} \int_{V}\left(\partial T/\partial x\right)dV & \int_{V}\left(\partial T/\partial y\right)dV & \int_{V}\left(\partial T/\partial z\right)dV \end{array}\right\}$$

(12) $$\int_{V}dV=V_{solid}+V_{air}$$

## 4 Boundary conditions

In order to evaluate the effective thermal conductivities using the constitutive law of the homogeneous medium, three different load cases need to be defined. One approach is to assume a fixed temperature gradient in the body in each load case, as described by Eqs. (13), (14), (15)

Load case 1:

(13) $${\bar{\nabla T}}^{\left(1\right)}=\left\{\begin{array}{c} g_{x}\\ 0\\ 0 \end{array}\right\}$$

Load case 2:

(14) $${\bar{\nabla T}}^{\left(2\right)}=\left\{\begin{array}{c} 0\\ g_{y}\\ 0 \end{array}\right\}$$

Load case 3:

(15) $${\bar{\nabla T}}^{\left(3\right)}=\left\{\begin{array}{c} 0\\ 0\\ g_{z} \end{array}\right\}$$

where (*g*_*i*_*, i=x,y,z*) is the magnitude of the corresponding temperature gradient. Dirichlet boundary conditions are utilized to impose a constant thermal gradient along a particular boundary. In this case the temperatures at two opposite faces of the RVE are given by Eq. (16):

(16) $$T\left(x_{1},x_{2},x_{3}\right)=T_{0}+\left({\bar{\nabla T}}^{\left(i\right)}\cdot {\vec{e}}_{i}\right)x_{i},i=1,2,3$$

The variables *x*_*1*_, *x*_*2*_, *x*_*3*_ correspond to the coordinate directions *x*, *y* and *z*, respectively and ${\vec{e}}_{i}$ is a unit vector in *x*_*i*_-direction. The scalar product term represents the magnitude of the temperature gradient along the *x*_*i*_-direction. This equation effectively assigns temperature values based on the predefined thermal gradient along the specified direction, where the temperature increases linearly with the distance along the direction of the gradient [64–66]. Furthermore, in each load case the average heat flux vector can be evaluated and substituted with the corresponding thermal gradient in Fourier's equation. This process leads to the following equations (17), (18), (19)

(17) $${\bar{q}}_{x}^{\left(1\right)}={\bar{k}}_{xx}g_{x}$$

(18) $${\bar{q}}_{y}^{\left(2\right)}={\bar{k}}_{yy}g_{y}$$

(19) $${\bar{q}}_{z}^{\left(3\right)}={\bar{k}}_{zz}g_{z}$$

The last three equations can be solved for the average thermal conductivities in the corresponding coordinate directions. To achieve this, the averaged of heat flux vector and temperature gradient need to be evaluated based on equations (9), (11). The heat flux and temperature fields are obtained by post-processing the results of the FEM analysis performed on the RVE. For accurate evaluation of the effective thermal characteristics of the homogenized unit cell for each load case a corresponding load path must to be created. The incremental load application provides the points on the load path. Furthermore, in the postprossecing at each loading level averaged variables are evaluated. These steps are controlled by a script file which is implemented in the software.

## 5 Model validation and proof of concept

The procedure described above is used to derive a homogeneous model of the RVE displayed in Fig. 3. Aluminum alloy is considered as the solid material with the data given in [**24**].

The following geometrical parameters are used.

➢ Side length of the hexagonal unit cell: 5 mm
➢ Cell angle: 60°
➢ Thickness in the z-direction: 10 mm

These characteristics result in a solid volume fraction of 0.02. The computed material parameters of the homogeneous unit cell are.

➢ Density: 55 kg/m³
➢ Specific heat capacity: 830 J/kg/°C
➢ Matrix of thermal conductivity:

$$\left[k^{eff}\right]=\left[\begin{array}{ccc} 1.3965 & 0 & 0\\ 0 & 1.389 & 0\\ 0 & 0 & 2.74 \end{array}\right]\;W/m/{}^{\circ}C$$

**Fig. 4:**
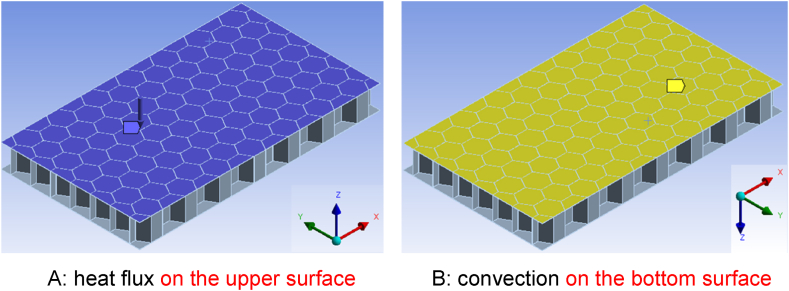
Boundary conditions.

To demonstrate the validity of the generated model, a numerical verification process is carried out of the thermal and mechanical homogenous model representing the honeycomb structure. The homogenized medium is discretized by an FE mesh with 16430 elements and 22464 nodes, while the real honeycomb has 494263 elements and 464965 nodes. For this purpose, the achieved effective material characteristics are used to describe the thermal and mechanical behavior of a homogeneous orthotropic structure. A honeycomb structure with the same geometrical dimensions is also created. In the thermal analysis both bodies are subjected to the same transient thermal boundary conditions. In the mechanical analysis the modal performance of both bodies is considered. In both investigations the achieved responses of the two structures are compared. To perform the thermal analysis the following boundary conditions are applied:

Time dependent heat flux on the upper surface and heat convection on the bottom surface, as shown in Fig. 4 (A, B). The other boundaries are assumed to be thermally insulated.

**Fig. 5:**
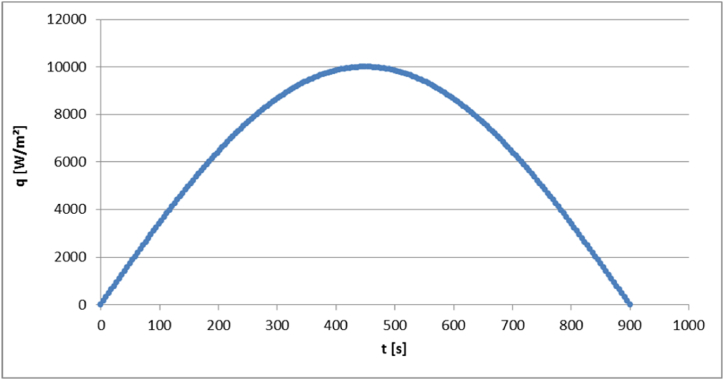
Heat flux variation with time.

The heat flux variation with time is represented by a half sine wave, as displayed in Fig. 5. The ambient temperature for heat convection is set to 22 °C and the heat transfer coefficient has a value of 8 W/m^2^/°C. These values are based on the data given in [**24**]. In the next section a deeper insight into the computational results of the analysis is given. The thermal performance of the homogenized continuous model is compared with the behavior of the original, detailed honeycomb structure. This allows for an evaluation of the system's overall performance and effectiveness and validates the presented approach. Additionally, these results form the basis for further discussions of the model's applications.

## 6 Validation of the results

**Fig. 6:**
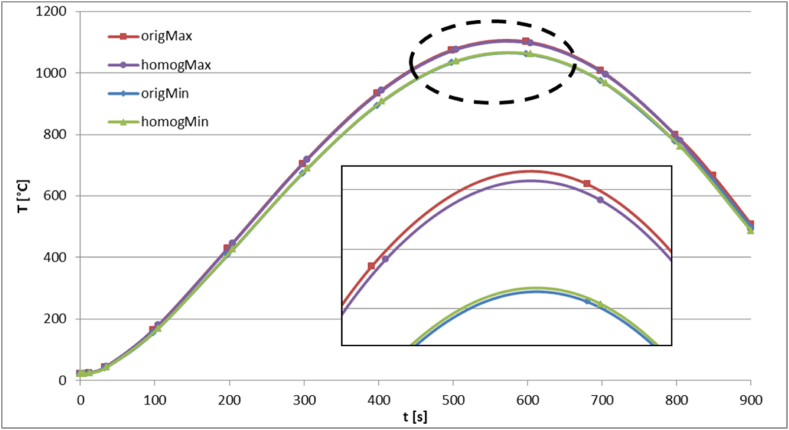
Evolution of minimum and maximum temperatures with time at the locations.

To compare the thermal behavior of the actual structure and the homogenous medium, the temperature evolution with time is compared in both bodies at locations of minimum and maximum temperatures, as displayed in Fig. 6. The locations correspond to the bottom and top surfaces, respectively.

of minimum and maximum in real and homogenized structures.

**Fig. 7:**
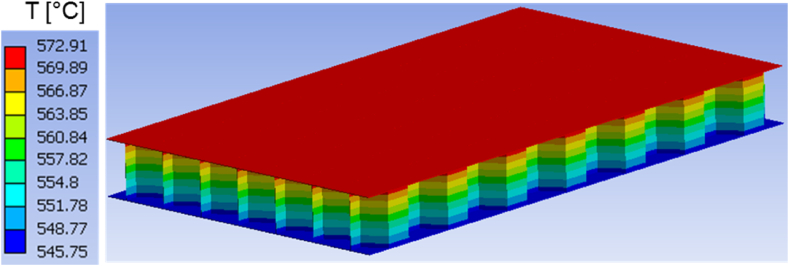
Temperature distribution at time t = 250 s in the honeycomb structure.

**Fig. 8:**
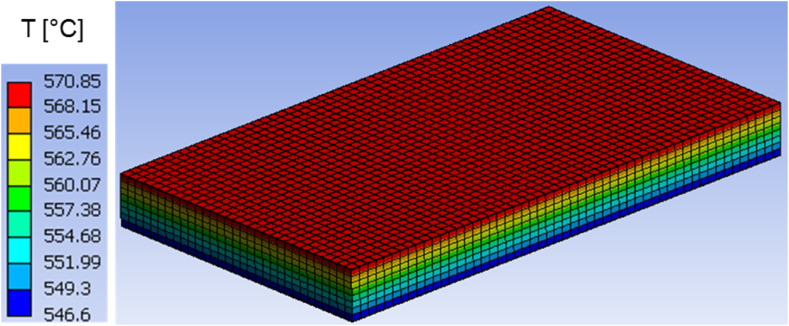
Temperature distribution at time t = 250 s in the homogeneous body.

**Fig. 9:**
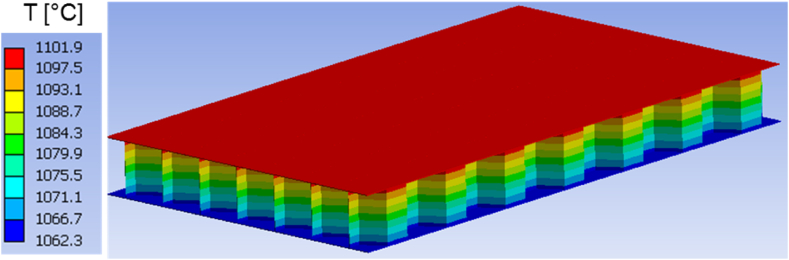
Temperature distribution at time t = 600 s in the honeycomb structure.

**Fig. 10:**
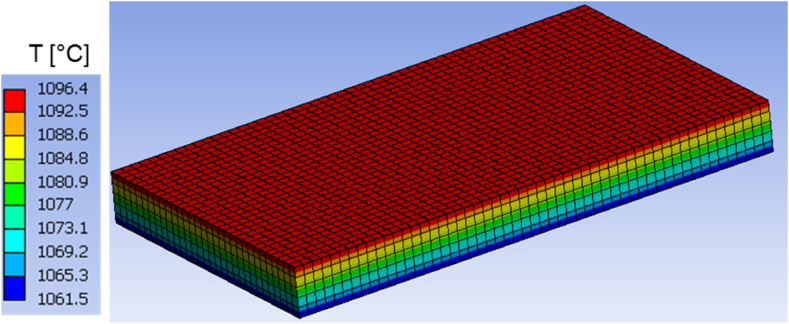
Temperature distribution at time t = 600 s in the homogeneous body.

**Fig. 11:**
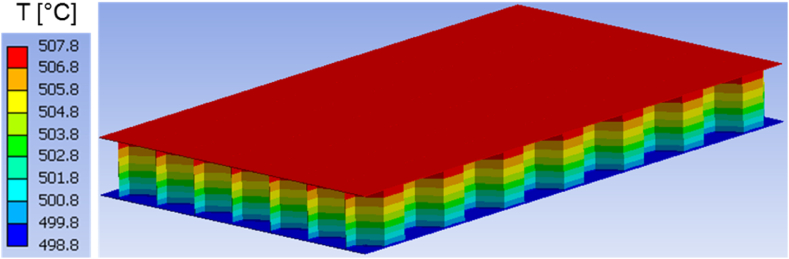
Temperature distribution at time t = 900 s in the honeycomb structure.

**Fig. 12:**
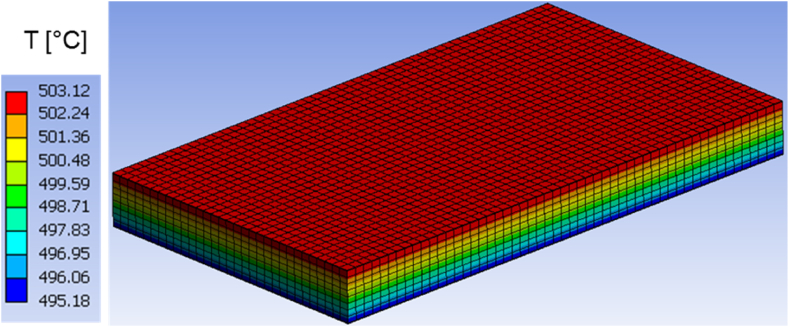
Temperature distribution at time t = 900 s in the homogeneous body.

Moreover, the predicted temperature fields in both structures are presented at different time instants in Fig. 7, Fig. 8, Fig. 9, Fig. 10, Fig. 11, Fig. 12. Clearly, the temperature profiles in both bodies show very close similarity over the entire time interval. At time instant 250 s, the temperature in the honeycomb structure is between 545.75 °C and 572.91 °C, as displayed in Fig. 7, Fig. 8. At the same time the temperature in the homogeneous structure reaches values between 546.56 °C and 570.74 °C. The temperature difference at the location of minimum is around less than 1 °C and at the location of maximum about 2 °C.

Around 600 s, when the external heat flux reaches its peak, the honeycomb structure exhibits temperatures ranging from 1062.3 °C to 1101.9 °C, as depicted in Fig. 9. The temperature in the homogenous medium, as shown in Fig. 10 reaches values between 1061.5 °C and 1096.4 °C at the same time. In regions of lowest and maximum, the temperature differences are respectively less than 1 °C and roughly 5.5 °C.

As the simulation time approaches its end, around 900 s, the temperature within the actual structure spans from 498.8 °C to 507.8 °C, as shown in Fig. 11. Simultaneously, the temperature within the homogeneous medium reaches values ranging from 495.18 °C to 503.12 °C. The variations between the highest and lowest temperatures in the corresponding bodies range between 3 °C and 4 °C, as presented in Fig. 12.

## 7 Model application

**Fig. 13:**
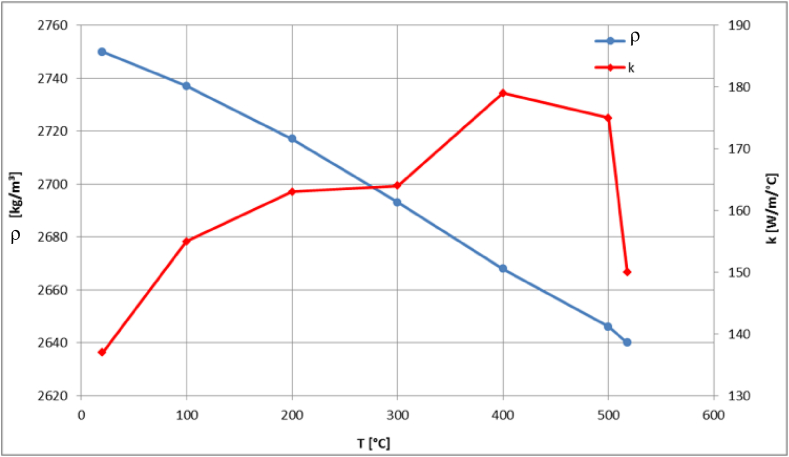
Aluminum's density (ρ) and thermal conductivity (k) as functions of temperature.

**Fig. 14:**
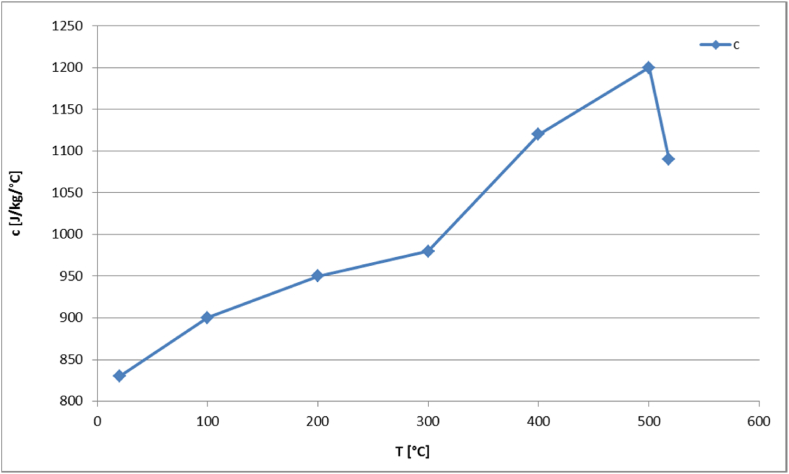
Aluminum's specific heat capacity (c) as function of temperature.

The generated homogeneous model is subjected to fire loading conditions. For this purpose, the temperature dependency of the thermo-physical properties of aluminum is considered, where the material data are taken from [61] and shown in Fig. 13, Fig. 14. As displayed in Fig. 13, the density of the material decreases with temperature. In the presented temperature interval between 25 °C and 520 °C the density falls from 2753 °C to 2492 °C. The thermal conductivity and specific heat capacity show an increasing behavior until the curve reaches the melting temperature and decreases as depicted in Fig. 13, Fig. 14, respectively.

**Fig. 15:**
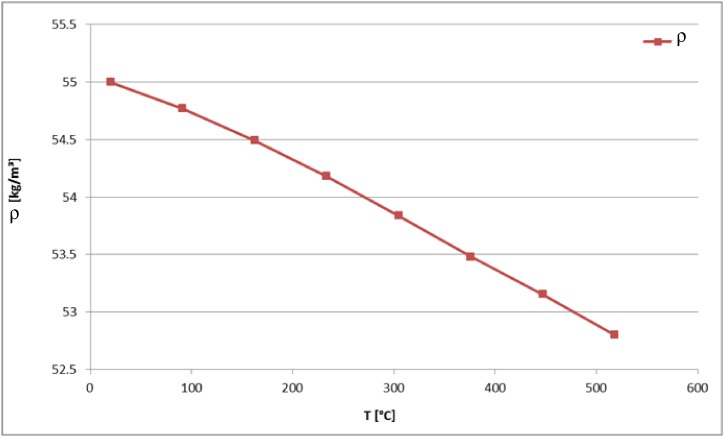
Density as function of temperature of the homogeneous medium.

**Fig. 16:**
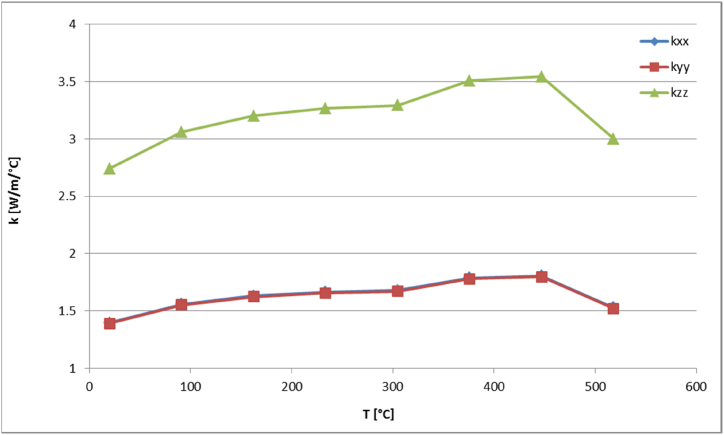
Thermal conductivities as function of temperature of the homogeneous medium.

**Fig. 17:**
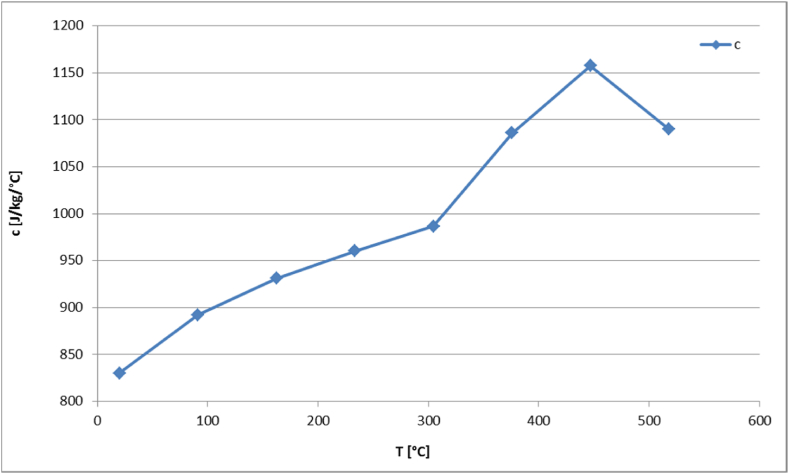
Specific heat capacity (c) as function of temperature of the homogeneous medium.

Based on the thermophysical properties of aluminum and using the RVE homogenization model, the characteristics of the homogeneous medium are evaluated as functions of time and displayed in Fig. 15, Fig. 16, Fig. 17. In general, the curves show a similar behavior to the honeycomb material. The density is falling with temperature, while the specific heat capacity rises until the melting temperature is reached. On the other hand the components of the thermal conductivity tensor show a smaller gradient with increasing temperature. The values of the homogenized material parameters are less than those of the honeycomb material due to the existence of large volume of void in the structure.

**Fig. 18:**
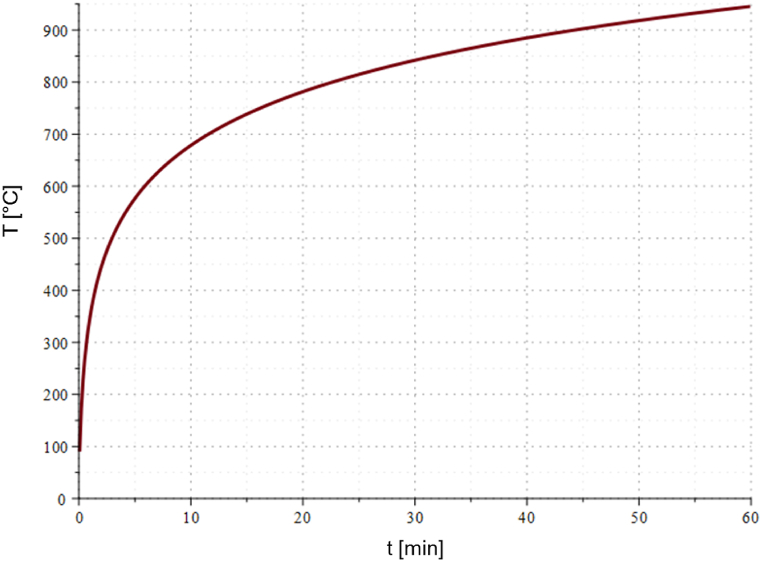
ISO 834 temperature-time curve.

For this investigation, an aluminum sandwich panel is used with geometrical dimensions 400 x 400 × 12.32 mm. The boundary conditions for heat transfer on the bottom and top surfaces of the panel are determined by convection. The ambient temperature on one side of the panel is determined by the ISO 834 standard temperature-time curve [62], while the ambient temperature on the other side is kept constant at 22 °C. The temperature curve is represented by Eq. (20) and illustrated in Fig. 18.

(20) T(t)=20C°+345*log10(8*t+1)C°

The temperature rises rapidly during the first 5 min of the process, which is evident, followed by a slower increase. At the end of the considered time period, the rate of temperature increase slows down, and the curve shows a tendency to converge.

**Fig. 19:**
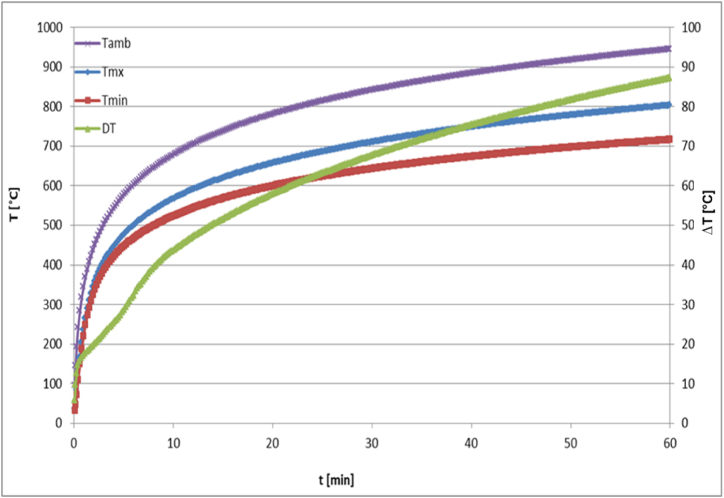
Variation of T_max_, T_min_ and ΔT with time.

**Fig. 20:**
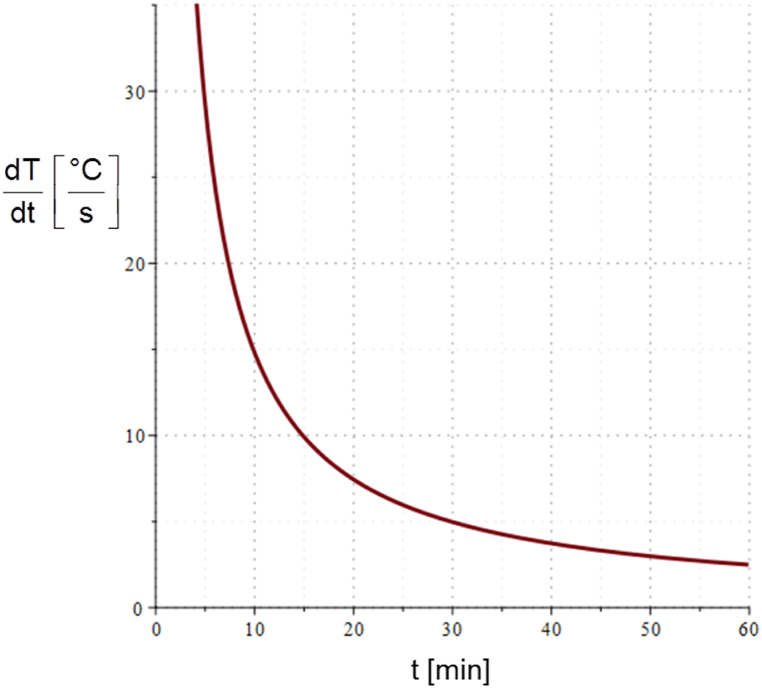
Variation of temperature rate of change with time.

Moreover, on both surfaces of the panel thermal radiation with the ambient is included in the analysis. In Fig. 19, the evolution of temperature at the maximum and minimum positions within the panel is displayed. These positions correspond to the upper and lower surfaces of the panel, respectively. The graph reveals how the temperature changes over time at these two distinct locations. One of the most important features to note is the difference in temperature between the upper and lower surfaces of the panel. This difference can be attributed to a number of factors such as exposure to the fire side with very high ambient temperature, heat conduction through the panel material, and convective heat exchange with the surrounding environment. The upper surface of the panel is more directly exposed to the fire ambient, resulting in a higher rate of heating compared to the lower surface. This difference in heating rate can be clearly observed in the trend of the temperature curves. As time progresses, the temperature difference between the two surfaces increases, indicating that the upper surface is being heated more rapidly than the lower surface. This is an indicator of the efficiency of using honeycomb structure for fire protection and reducing the transfer of heat. Furthermore, the curves are compared to the ambient ISO 834 temperature-time curve, as displayed in the figure. The three curves have comparable behavior; they increase rapidly at the beginning and slow down during the process and show a converging trend at the end. This behavior is depicted in the variation of temperature rate of change with time as illustrated in Fig. 20, where the curve converges to zero after a long time. The difference between the maximum and minimum temperatures (*ΔT*) rises with time, as presented in Fig. 19. After 60 min, at the end of the considered time period, *ΔT* reaches a value close to 90 °C.

**Fig. 21:**
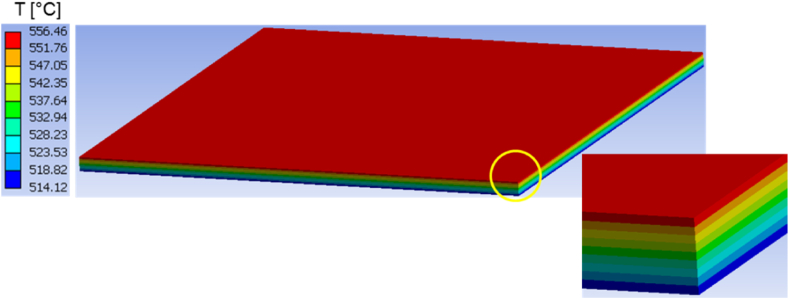
Temperature profile in the panel at T = 10 min.

In Fig. 21 the temperature profile in the panel is displayed at time instant around 10 min after applying the thermal load. The maximum ambient temperature reached is over *550 °C*, which is close to the solidus temperature of the considered material. At this time instant, the difference between maximum and minimum temperatures reached in the body is about *42 °C*.

**Fig. 22:**
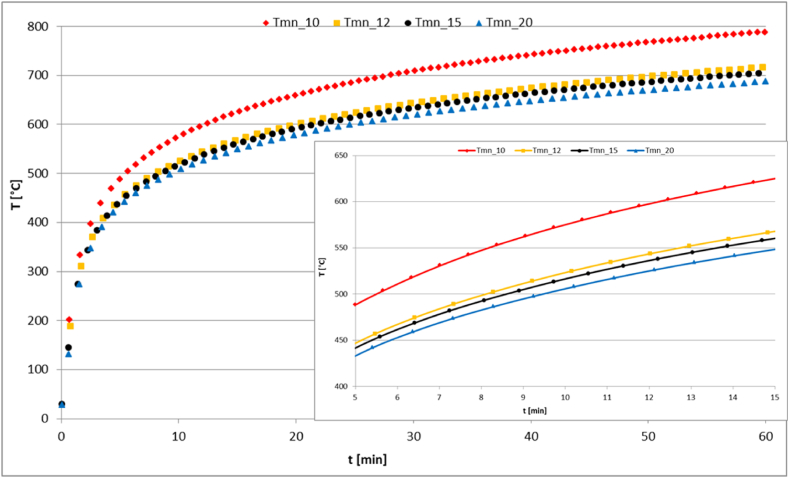
Variation of temperature with time and thickness on the lower surface of the panel.

Moreover, the effect of varying the panel thickness on the thermal response to the applied temperature load is investigated. The thickness of an aluminum honeycomb panel plays a crucial role in determining its thermal performance and overall efficiency. Varying the thickness can significantly impact the panel's ability to regulate heat transfer. Thicker panels tend to offer better thermal insulation, as they create a larger air gap within the honeycomb structure. This increased gap acts as a barrier to heat flow, raising the thermal resistance and minimizing heat transfer between the panel's surfaces. The thermal response of the panel is characterized by the minimum temperature, which occurs at the lower surface in Fig. 21. The temperature variation with time at the panel's bottom surface for the various thicknesses is depicted in Fig. 22. Obviously, with rising thickness the thermal resistance also increases, leading to reduction in the minimum temperature. This behavior is elucidated in the zoomed part of the curves in the time interval between 5 and 15 min.

Consequently, thicker panels are more effective at maintaining desired temperatures, providing enhanced thermal insulation and energy efficiency. Conversely, thinner panels have a smaller air gap, resulting in higher thermal conductivity and increased heat transfer. While they may be suitable for applications that require heat dissipation or rapid temperature response, thinner panels generally offer lower thermal insulation. Thus, selecting the appropriate thickness for an aluminum honeycomb panel is crucial for achieving the desired thermal performance in various applications.

## 8 Modal behavior of the homogenized structure

**Fig. 23:**
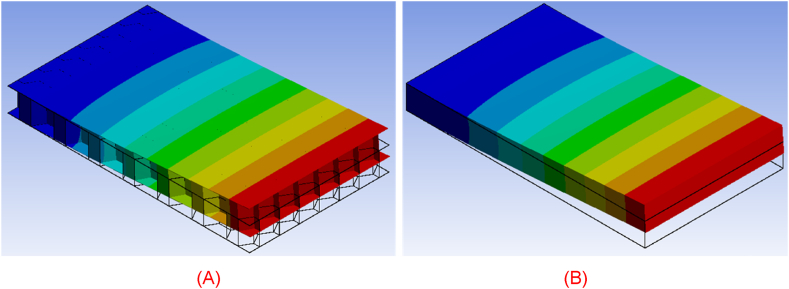
(A) First natural vibration mode of honeycomb structure and (B) homogenized medium.

**Fig. 24:**
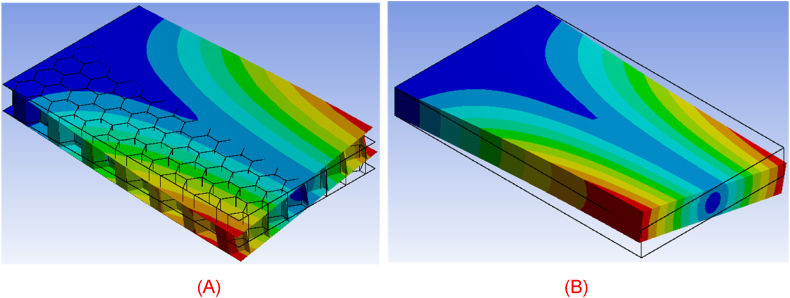
(A) Second natural vibration mode of honeycomb structure and (B) homogenized medium.

**Fig. 25:**
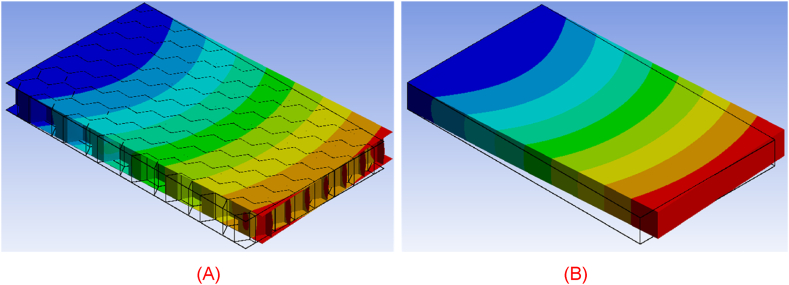
(A) Third natural vibration mode of honeycomb structure and (B) homogenized medium.

**Fig. 26:**
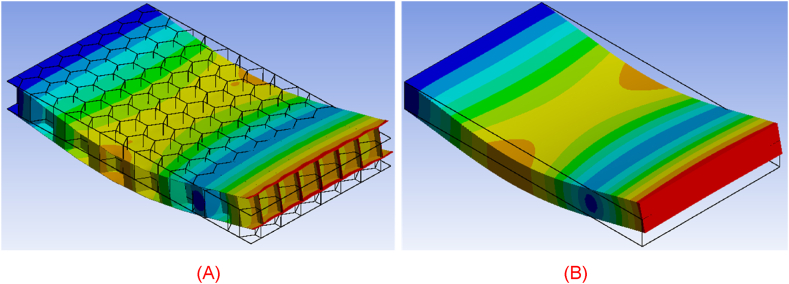
(A) Fourth natural vibration mode of honeycomb structure and (B) homogenized medium.

As mentioned previously, the modal response of the modeling approach is also investigated. The comparison of the two cases is demonstrated by investigating the first four natural modes of vibration of the real honeycomb structure and its homogenized representative model. As displayed in Fig. 23, Fig. 24, Fig. 25, Fig. 26, the deformation of both bodies shows a very good agreement in the four cases. The correlation between the modal response and natural frequencies between the real honeycomb structure and the homogenized model is remarkably strong, highlighting the effectiveness of the homogenization technique. The modal response, which characterizes the dynamic behavior of a system, reflects how it vibrates in different modes of oscillation. When comparing the modal responses of a real model and a homogenized model, a striking similarity is observed in terms of their shapes and patterns. This agreement suggests that the homogenized model accurately captures the essential dynamic behavior of the real system.

**Fig. 27:**
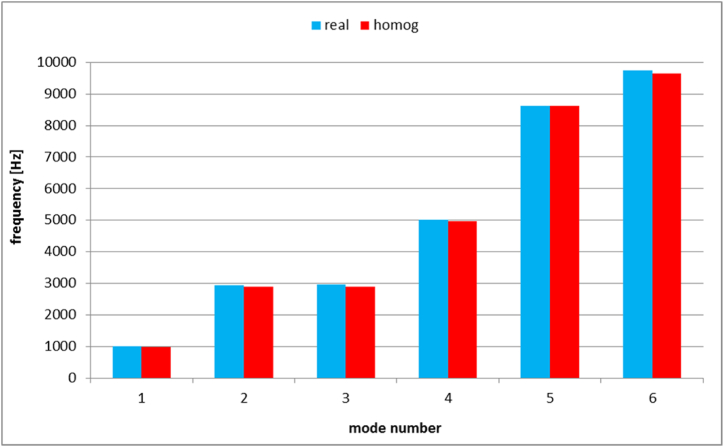
Natural frequencies of honeycomb structure and homogeneous body.

Furthermore, the natural frequencies, which correspond to the frequencies at which the system naturally vibrates, exhibit a close alignment between the real model and the homogenized model which can be clearly seen in Fig. 27 and in Table 1. Within the range of natural frequencies examined, which spans from 1013.8 Hz to 9735.8 Hz, the disparity in response between the actual honeycomb structure and the uniform medium falls between 0 Hz and 72 Hz. This congruence not only validates the homogenization approach but also enhances its utility in predicting and analyzing the dynamic responses of complex structures with confidence and efficiency.

**Table 1:** natural frequencies.

Mode number	1	2	3	4	5	6
Honeycomb structure [Hz]	1013.8	2931.6	2971.8	5021.7	8624.8	9735.8
Homogenized medium [Hz]	985.5	2892.3	2899.9	4972.2	8624.8	9640.4

## 9 Conclusions

In summary, this research focused on the application of homogenization, employing mathematical techniques to determine effective properties of heterogeneous materials at a macroscopic scale. Utilizing common tools such as RVE and FEM, the thermal performance of aluminum-core honeycomb structures was explored. The analysis involved solving the heat conduction problem numerically through FEM and creating a computational FE domain based on an appropriate RVE. The orthotropic nature of the honeycomb structure was considered, leading to the development of an effective orthotropic thermal conductivity tensor that characterizes the behavior of the homogenized medium. Temperature-dependent and nonlinear material characteristics were incorporated to assess thermal responses under different boundary conditions.

To validate the model's accuracy, a numerical verification process compared the thermal and mechanical properties of the homogenized model to the actual honeycomb structure. While the discretized homogenized medium used an FE mesh with 16430 elements and 22464 nodes, the real honeycomb had significantly more elements and nodes. The results demonstrated a strong correlation between the two structures, particularly in thermal responses under applied boundary conditions. The study further explored the effect of varying panel thickness on thermal performance. Thicker panels were found to offer enhanced thermal insulation and energy efficiency due to their larger air gap, while thinner panels allowed for increased heat transfer and were suitable for applications requiring rapid temperature response or heat dissipation. In terms of mechanical properties, the structural modal behavior of the honeycomb structure and the homogenized medium were compared within a range of natural frequencies. The difference in responses was found to be within a specific range of frequencies.

In conclusion, this research provides valuable insights into the thermal and mechanical properties of aluminum honeycomb structures using homogenization techniques, shedding light on their thermal insulation capabilities and structural modal responses. These findings have practical implications for selecting the appropriate panel thickness in various applications to achieve the desired thermal performance and efficiency.

## Data availability statement

Data will be made available on request.

## CRediT authorship contribution statement

**Ali Al-Masri:** Writing – review & editing, Validation, Investigation, Formal analysis. **Khalil Khanafer:** Writing – review & editing, Formal analysis. **Kambiz Vafai:** Writing – review & editing, Supervision, Conceptualization.

## Declaration of competing interest

The authors declare that they have no known competing financial interests or personal relationships that could have appeared to influence the work reported in this paper.
